# Assessment of Peripheral Airway Function following Chronic Allergen Challenge in a Sheep Model of Asthma

**DOI:** 10.1371/journal.pone.0028740

**Published:** 2011-12-12

**Authors:** Joanne Van der Velden, Donna Barker, Garry Barcham, Emmanuel Koumoundouros, Kenneth Snibson

**Affiliations:** 1 Centre for Animal Biotechnology, Veterinary Science, University of Melbourne, Melbourne, Victoria, Australia; 2 School of Engineering, University of Melbourne, Melbourne, Victoria, Australia; Statens Serum Institute, Denmark

## Abstract

**Background:**

There is increasing evidence that the small airways contribute significantly to the pathophysiology of asthma. However, due to the difficulty in accessing distal lung regions in clinical settings, functional changes in the peripheral airways are often overlooked in studies of asthmatic patients. The aim of the current study was to characterize progressive changes in small airway function in sheep repeatedly challenged with house dust mite (HDM) allergen.

**Methodology/Principal Findings:**

Four spatially separate lung segments were utilized for HDM challenges. The right apical, right medial, right caudal and left caudal lung segments received 0, 8, 16 and 24 weekly challenges with HDM respectively. A wedged-bronchoscope technique was used to assess changes in peripheral resistance (R_p_) at rest, and in response to specific and non-specific stimuli throughout the trial. Allergen induced inflammatory cell infiltration into bronchoalveolar lavage and increases in R_p_ in response to HDM and methacholine were localized to treated lung segments, with no changes observed in adjacent lung segments. The acute response to HDM was variable between sheep, and was significantly correlated to airway responsiveness to methacholine (r_s_ = 0.095, P<0.01). There was no correlation between resting R_p_ and the number of weeks of HDM exposure. Nor was there a correlation between the magnitude of early-phase airway response and the number of HDM-challenges.

**Conclusions:**

Our findings indicate that airway responses to allergic and non-allergic stimuli are localized to specific treated areas of the lung. Furthermore, while there was a decline in peripheral airway function with HDM exposure, this decrease was not correlated with the length of allergen challenge.

## Introduction

Asthma is a chronic inflammatory disease involving both the proximal and distal airways. However, much of the current knowledge relating to functional changes in asthma is derived from studies examining the large airways. Consequently, the contribution of the small airways to asthma pathophysiology is not fully understood. There is increasing evidence of chronic inflammation and the presence of structural changes within the small airways of asthmatic patients [1], [2], [3]. In some cases, these changes are even more severe than what is seen in the large central airways [1]. However, as the resistance of the peripheral airways (R_p_) accounts for only a small fraction of total airway resistance, changes in small airway function are not readily detected using routine pulmonary function testing [2].

To date, research in this area has been hampered by the difficulty of accessing the peripheral airways in routine lung function testing in the clinic [3]. Progress has been made through recent developments in imaging techniques, such as high resolution computed tomography (HRCT) and magnetic resonance imaging (MRI), which allow for the visualization of airway dimensions in airways as small as 2 mm [4]. However, while these techniques have the benefit of being non-invasive, they only offer indirect measurements of small airway function. Peripheral airway mechanics can, however, be assessed directly using either the forced oscillation technique (FOT) or wedged-bronchoscope technique [5], [6], [7]. FOT involves applying low frequency oscillations of pressure at the airway opening during normal breathing [8], while the wedged bronchoscope technique is performed by passing a constant airflow into a lung segment via a bronchoscope wedged in an airway of interest and measuring changes in pressure at the bronchoscope tip to calculate R_p_; i.e. pressure/flow.

The study of small airway function in asthma can benefit from the use of animal models where disease conditions can be easily controlled and manipulated. Large animals can be particularly useful for studies investigating changes in peripheral airway function in asthma because their small airways share many similarities with human small airways, including: an extensive bronchial wall microcirculation, an active mast cell component and the presence of mucous glands and smooth muscle [9]. These features, which play significant roles in the pathophysiological changes in asthma, are either absent or poorly represented in the peripheral airways of the mouse, which are by far the most popular species used to model allergic airways disease [10]. Importantly, in sheep, lung function can be measured in awake, spontaneously breathing animals without any confounding effects of anesthetics.

The aim of the current study was to use the sheep model of asthma to investigate changes in small airway function in response to chronic exposure to a relevant human allergen, house dust mite (HDM), using a segmental challenge regime. First, the wedged-bronchoscope technique was used to determine whether airway responses to allergic and non-allergic stimuli are localized to specifically targeted lung segments. Progressive changes in small airway function were then characterized in sheep in four lung segments treated with HDM allergen different durations.

## Materials and Methods

### Ethics Statement

All experimental animal procedures and the collection of tissues/cells were approved by the Animal Experimentation Ethics Committee of the University of Melbourne (approval no. 06128).

### Experimental Animals and Allergen Sensitization

Fourteen female Merino-cross sheep (6 months) were immunized subcutaneously with HDM extract (*Dermatophagoides pteronyssins*; CLS, Melbourne, Australia) as described previously [11]. Pre- and post-immunization HDM-specific serum IgE levels were assessed be ELISA [12] and sheep with at least a 2-fold increase were considered atopic and selected for further HDM-challenges. A fibre-optic bronchoscope was used for the localized delivery of solubilized HDM (1 mg in 5 ml phosphate buffered saline [PBS]) into individual lung segments.

### Measurements of Peripheral Airways Resistance

**Figure 1:**
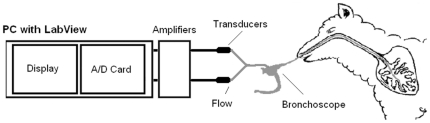
Schematic diagram showing segmental physiology setup. A stream of 5% CO_2_ in air was passed through the biopsy port of the bronchoscope wedged in a lung segment. Pressure and flow data were collected via an A/D card and analyzed with LabVIEW® software.

R_p_ was measured in individual lung segments of awake, consciously breathing sheep using a wedged-bronchoscope technique similar to a previously described procedure [7] and a custom built Airway Monitoring System. In particular, to measure R_p_ a controlled flow (6 mL/s) of 5% CO_2_ in air was passed through the working channel of a bronchoscope wedged into an airway in the lung segment of interest (Figure 1). Both pressure and flow in the wedged segment were measured continuously at the proximal end of the bronchoscope via tubing and T-piece connector which were fitted into the biopsy port of the endoscope. A T-series transducer (Gaeltec Ltd, Dunvegan, Scotland) measured pressure and a Series 820 mass flow meter (SIERRA Instruments Inc., Monterey, USA) measured inline flow. The pressure and flow transducers were connected to PM-1000 Transducer Amplifiers (CWE Inc., Admore, USA). Data was acquired with Data Acquisition Card (PCI-6233; National Instruments Corp., Austin, USA) and was analyzed with customized Labview Pty Ltd software.

R_p_ was calculated as mean pressure/flow during tidal breathing when pressure had stabilized and flow adjusted to 6 ml/s. The measurement of R_p_ in the wedged segment usually only took a minute to complete. All resistance measurements were corrected for the resistance of flow through the biopsy channel of the bronchoscope.

### Airway Responses to Allergic and Non-allergic Stimuli

The early-phase airway response (EAR) to HDM was measured in individual lung segments by monitoring the change in R_p_ at 15 minute intervals for 60 minutes post-challenge and calculating the percent change from baseline. The peak of allergen-induced bronchoconstriction typically occurs within 60 minute in this model [13].

To assess airway responses to methacholine a nebulizing catheter was passed through the bronchoscope. Following an initial delivery of PBS, doubling doses of methacholine (Sigma-Aldrich, Stenhein, Germany) were nebulized directly into the segment (0.009, 0.018, 0.037, 0.075 w/v) for 30 seconds. R_p_ was measured after each delivery and the accumulative dose of methacholine needed to increase the baseline R_p_ by 100% (PC_100_) was calculated.

### Bronchoalveolar Lavage Collection

Bronchoalveolar lavage (BAL) was collected from individual lung segments via slow infusion and withdrawal of 10 mL of sterile PBS through the working channel of the bronchoscope. The total number of cells collected was determined using a haemocytometer. Differential leukocyte counts were performed on cytospots of BAL cells stained with Haem Kwik Differential Stains (HD Scientific, Wetherill Park, Australia) to identify eosinophils.

### Statistical Analysis

Data are presented as means ± SEM. A Wilcoxon signed-rank test was used to compare between two lung segments within one group. A one-way ANOVA was used to compare between three or more segments. A Mann-Whitney test was used to compare between control and HDM challenged sheep groups. Correlations were assessed by the Spearman correlation coefficient (r_s_). A P value of <0.05 was considered significant. Statistical analysis was performed using GraphPad Prism for Windows (GraphPad Software Inc., La Jolla, USA).

## Results

### Localization of Airway Responses in Spatially Separate Lung Segments

**Figure 2:**
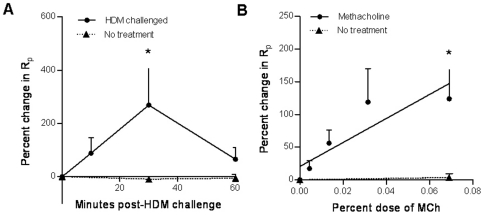
Peripheral airway responses to allergic and non-allergic stimuli are isolated to treated lung segments. Measurements from two spatially separate lung segments in sheep sensitized to house dust mite (HDM). (*a*) Percent change in peripheral airway resistance (R_p_) from the resistance after saline following either HDM challenge or no treatment. (*b*) Percent change in R_p_ from saline following increasing doses of methacholine (MCh) or no treatment. The doses of methacholine represented on the x-axis are the percentages of methacholine w/v administered to the lung segment via a bronchoscopic nebulising catheter as a 30 s nebulised aerosol. Mean+SEM, N = 7, *P<0.05 compared to no treatment.

To test whether peripheral airway responses were localized to spatially separate lung regions, R_p_ was assessed in two spatially separate lung segment in the same sheep which received a HDM challenge or no treatment, in the left and right caudal respectively (n = 7). R_p_ was measured immediately prior to HDM challenge (0 min) and at 15 minute intervals for 60 minutes post-challenge in each segment. The R_p_ in the HDM-challenged segment increased by 268±178% from baseline (Figure 2A), while there was no change in the R_p_ in the adjacent untreated lung segment (−9.7±4.6%).

Assessment of airway responsiveness to methacholine also showed that changes in R_p_ were isolated to treated lung segments; i.e. delivery of 0.043% methacholine was required for PC_100_ in the left caudal segment, while there was no change in R_p_ in the right caudal lung segment, which was untreated (Figure 2B).

**Figure 3:**
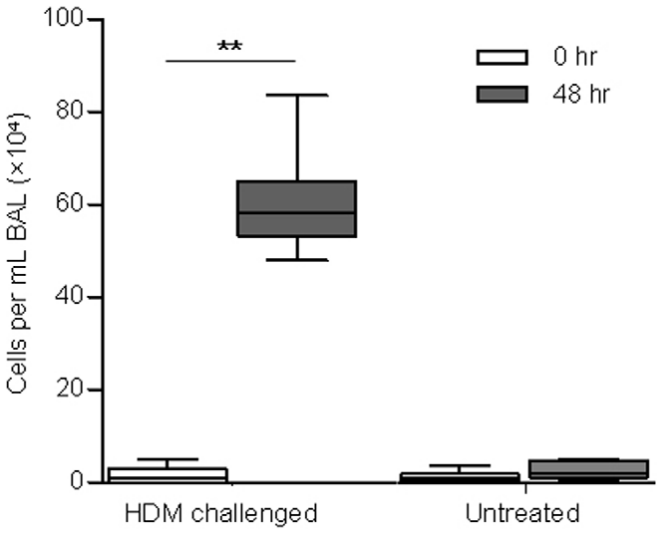
Recruitment of eosinophils into bronchoalveolar lavage was isolated to HDM treated lung regions. Graph shows the number of eosinophils per mL of bronchoalveolar lavage (BAL) cells from two spatially separate lung segments which received either a house dust mite (HDM) challenge or no treatment. BAL was collected at baseline (0 hrs) and 48 hrs following a HDM challenge. Horizontal lines represent the mean, boxes represent the 25^th^ and 75^th^ percentiles, whiskers represent the 5^th^ and 95^th^ percentiles. N = 8, **P<0.01 compared to 0 hrs.

The number of eosinophils in BAL was assessed 48 hours following allergen exposure in the treated and untreated segments and compared to pre-challenge levels. As expected, there was a significant increase in the number of eosinophils per mL of BAL in the HDM-challenged segment 48 hrs post-challenge compared to baseline (2.6±1.0×10^3^ vs. 1.3±0.3×10^6^ cells per mL, P<0.01; Figure 3). However, there was no difference in the percent of eosinophils present in BAL between 0 and 48 hrs in the untreated segment (2.1±1.0×10^3^ vs. 6.7±1.6×10^3^ cells per mL, P = 0.15; Figure 3).

### Changes in Peripheral Airway Function Following Chronic HDM Challenges

The reliability of using the wedged-bronchoscope technique to measure R_p_ was assessed by recording two separate measurements of R_p_ from the right caudal lung segment of untreated sheep, two weeks apart. The coefficient of variation (C_v_) for the repeated measures was 9±2 (Table S1), which is similar to previously published C_v_ values for repeated measurements of total airway resistance [14].

To determine whether there were progressive changes in peripheral airway function, four spatially separate lung segments were identified within sheep and utilized for repeated HDM challenges. The duration of HDM exposure was staggered in each of the segments so that at the end of the trial, the right medial, right caudal and left caudal segments received a total of 8, 16 or 24 weekly challenges with HDM respectively (Figure S1). The right apical lobe was utilized as an untreated control segment, as our previous studies have that shown long term challenges with saline has no effect on lung function or airway responsiveness to non-specific stimuli [13].

**Figure 4:**
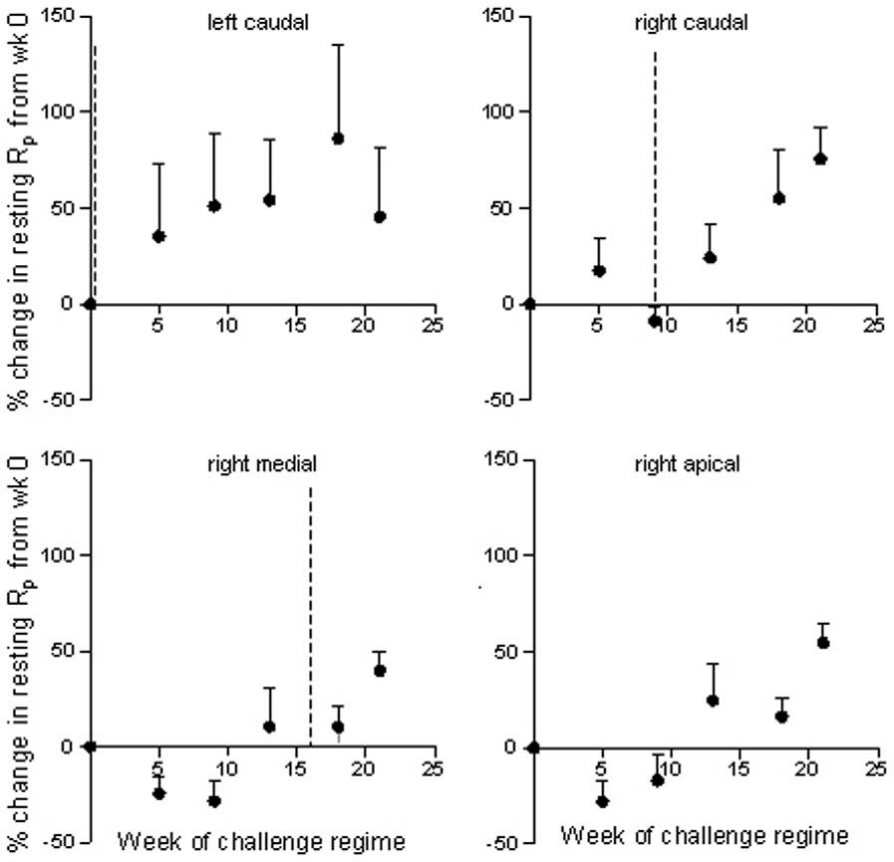
Changes in resting peripheral resistance following chronic allergen challenge. Graphs show the percent change in resting peripheral airway resistance (R_p_) in four spatially separate lung segments. House dust mite (HDM) challenges in each of the segments were staggered so that the right apical, right medial, right caudal and left caudal lung segments received a total of 0, 8, 16 or 24 challenges respectively. The dotted lines represent the commencement of challenges in each segment. Mean+SEM. n = 7.

Resting R_p_ was assessed in each segment prior to the commencement of HDM challenges and throughout the challenge regime. Interestingly there was an increase in resting R_p_ which corresponded with the commencement of HDM challenges in each segment (Figure 4). However, there was no correlation between the percent change in resting R_p_ from week 0 and the number of HDM challenges received (r_s_ = 0.657, P = 0.175).

**Figure 5:**
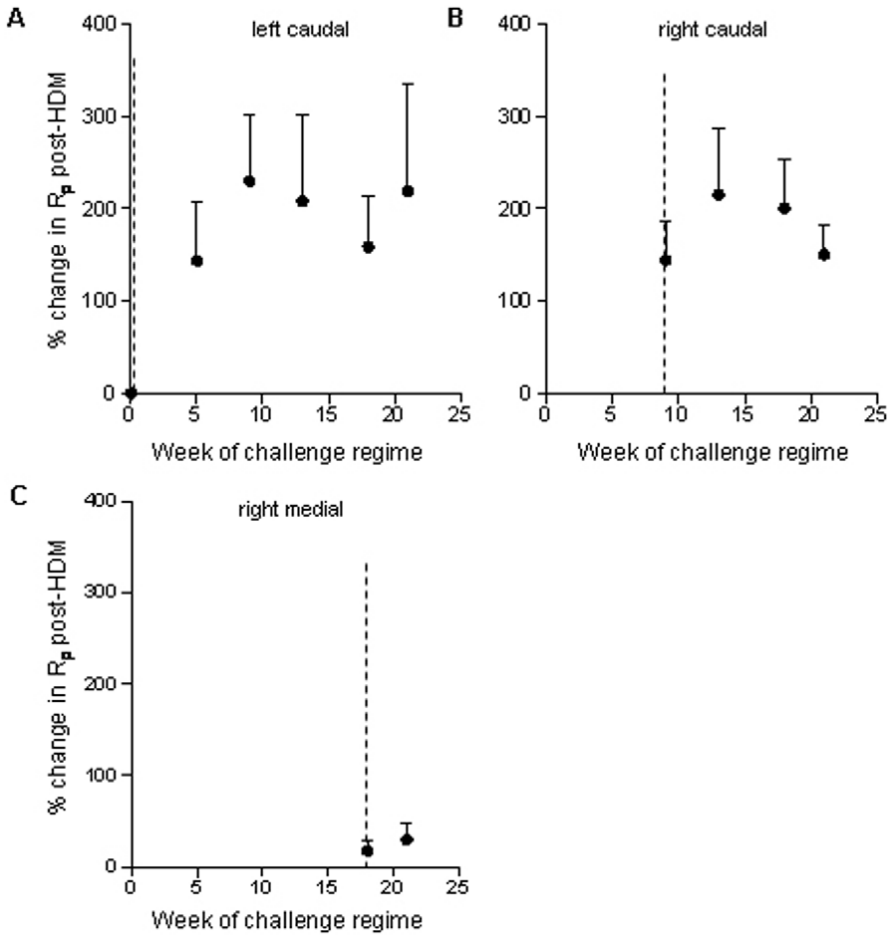
Early-phase asthmatic responses in spatially separate lung segments following allergen challenge. The percent change in peripheral airway resistance (R_p_) 30 min post-house dust mite (HDM) challenge from resistance four spatially separate lung segments (right apical, right medial, right caudal and left caudal). The numbers of the x-axis indicate the number of HDM challenges that segment has received at week 9, 13, 18 and 21. Mean + SEM. n = 7, *P<0.05.

Early airway responses (EAR) to HDM were assessed at weeks 9, 13, 18 and 21 of the challenge protocol. There was a natural variation in local lung function changes between individual sheep in the HDM-challenge group at each of the time-points. The percent increase in R_p_ from rest within the first hour following HDM challenge at week 21 ranged from 32 to 910%. Surprisingly, there was no progressive increase in the magnitude of EAR with increasing exposure to HDM. Instead, there appeared to be a plateau in the EAR, with the mean percent increase in the R_p_ of the left caudal lung segment remaining at around 220% at week 9, 13, 18 and 21 despite there being a progressive increase in the number of HDM challenges at these time-points (Figure 5).

**Figure 6:**
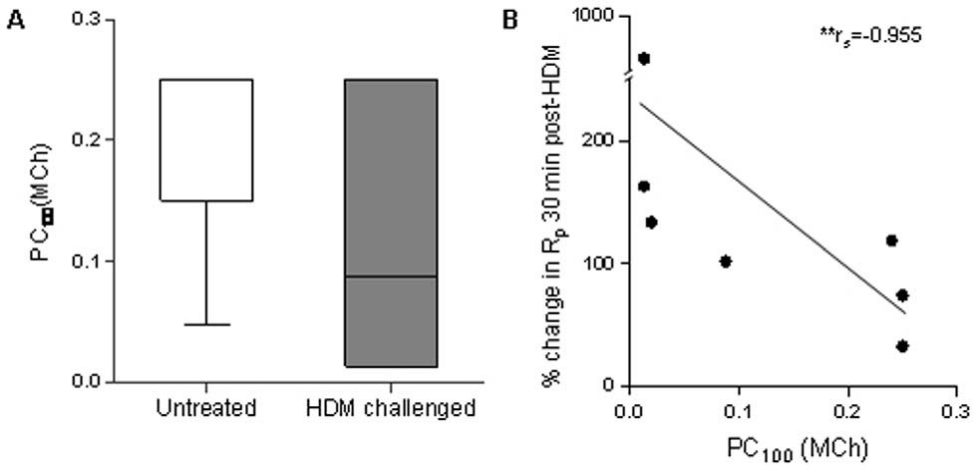
Changes in peripheral airway responsiveness to methacholine following chronic allergen challenge. (*a*) Percent dose of methacholine required to raise the peripheral airway resistance (R_p_) 100% from the resistance after saline (PC_100_[MCh]) for an untreated control lung segment and a lung segment chronically challenged with house dust mite (HDM) for 23 weeks. Horizontal lines represent the mean, boxes represent the 25^th^ and 75^th^ percentiles, whiskers represent the 5^th^ and 95^th^ percentiles. (*b*) Correlation between PC_100_(MCh) and the percent change in R_p_ from saline 30 min post-HDM challenge (r_s_ = −0.955) assessed between weeks 21–23. n = 7, **P<0.01.

The doses of methacholine for PC_100_ in the left caudal segment of the HDM-challenged group ranged between 0.012 to 0.250%. Although the difference was not significant, there was a trend the HDM-challenged segment to require a lower dose of methacholine for PC_100_ compared to an untreated segment in a separate group of control sheep (0.12±0.04 vs. 0.20±0.03, P = 0.18; Figure 6A). There was a significant negative correlation between the dose of methacholine for PC_100_ and EAR (r_s_ = −0.955, P<0.01; Figure 6B); i.e. individuals with highly responsive airways to non-specific stimuli also had high acute-allergen induced increases in resistance. A correlation analysis showed there was no significant correlation between the percent change in resting R_p_ and airway responsiveness (r_s_ = −0.433, P = 0.354). Nor was there any correlation between resting R_p_ and EAR (r_s_ = 0.393, P = 0.396).

## Discussion

In the work presented here, HDM and methacholine were shown only to affect peripheral resistance in the treated lung regions, without affecting adjacent, untreated, segments in the same sheep. Importantly, repeated bronchoscopy had no effect on subsequent resistance measurements in the untreated segment, demonstrating that increases in R_p_ in the treated segment were a result of the airway challenges with HDM and methacholine, and not due to repeated sampling. While previous studies in sheep have demonstrated that structural airway remodeling is localized to regions of HDM delivery [15], to our knowledge this is the first evidence that changes in airway function are also confined to the sites of allergen challenge. Importantly, it was also found that infiltration of eosinophils into the lungs following an allergen challenge was localized to the site of HDM delivery. The number of eosinophils per mL of BAL was unchanged in the untreated lung segments, while there was a significant increase in HDM-treated segments, indicating that there was no ‘spill-over’ of HDM between adjacent lung segments in the same animal. The advantage of comparing different treatments in one animal is that the influences of genetic background normally found between sheep are negated and hence variability is reduced. In addition, experiments designed to utilize the segmental treatment approach require less animals than whole lung experiments providing both ethical and financial benefits.

The resting resistance values reported here, are very similar to previously published values for sheep [6] and humans [16], [17]. Importantly, in the current study there was little variation between repeat measurements of resting R_p_ indicating that resistance measurements collected using the wedged-bronchoscope method are reliable. The low level of variability between measurements in this study also means that any changes in R_p_ in the small airways in response to allergen exposure should readily be detected using this method.

While there was an initial increase in resting R_p_ and in the magnitude of EAR with the commencement of HDM challenges, both indices of lung function stabilized after several weeks, despite continuing HDM challenges. This plateau could potentially be explained by increases in the extracellular matrix surrounding the airways, which may act to stiffen the airway walls and prevent them from collapse, thus protecting the airways against lung function decline. Previous studies have shown that there are significant increases in the amount of collagen and fibronectin surrounding the airway walls of Brown-Norwegian rats following increasing exposure to HDM allergen, and that this was associated with improvements in lung function [18], [19]. While structural changes within the airway wall were not assessed in the current study, our previous studies using this sheep model have shown that collagen is significantly increased in sheep challenged with HDM for 25 weeks.

The variation in functional responses between sheep in the current study has been previously reported in this [13] and other [20] sheep models of asthma. It is likely that the wide variation observed in this study reflects a separation of sheep into ‘responders’ and ‘non-responders’, based on their functional responses to stimuli, as identified in previous studies [13], [20], [21]. This hypothesis is supported by the correlation between segmental airway responsiveness and EAR in HDM-challenged sheep, in that individuals which had highly responsive airways to methacholine also had high EAR. Conversely, sheep which showed low responsiveness to methacholine also showed little change in R_p_ following HDM challenge. Unlike typical laboratory mice, which are commonly used to model allergic airway disease, sheep are an outbred species, and thus there can be a continuum in the range of functional responses between individuals due to genetic differences between individuals. The range of responses is similar to that observed in humans. In any case, the variable responses to stimuli will allow us to investigate which pathological airway changes may be associated with changes in lung function in future experiments.

In summary, the results presented here indicate that the segmental challenge model of asthma will be useful for investigating changes in the small airways. In particular, the segmental challenge model presented here could be used to correlate structural and inflammatory cell changes in the small airways with functional changes, as these relationships due to a number of ethical and logistical constrains cannot easily be assessed in asthmatic subjects. A better understanding of small airway function in asthma could lead to new formulations of inhaled therapies with a smaller particle size, which may improve delivery to peripheral regions of the lung [3].

## Supporting Information

Figure S1 **Schematic diagram showing the protocol for segmental allergen challenge in four spatially separate lung segments.** (*a*) The right apical, right medial, right caudal and left caudal segments received 0, 8, 16 and 24 weekly infusions of 1 mg house dust mite allergen in 5 mL of phosphate buffered saline. (*b*) Schematic diagram of a sheep lung showing the location of each lung segment.
(TIF)

Table S1 **Repeated measures of resting peripheral resistance.** Repeated measurements of the resting peripheral resistance (R_p_) in individual sheep collected two weeks apart. Units are expressed in cmH_2_O/L/min. Repeated measures were collected two weeks apart. C_v_ – coefficient of variation. SEM – standard error of the mean.
(TIF)
