# Supplementary figures and images for: Assessment of Peripheral Airway Function following Chronic Allergen Challenge in a Sheep Model of Asthma
Source: PLoS One. 2011 Dec 12;6(12):e28740. doi: 10.1371/journal.pone.0028740 (PMC3236205; doi:10.1371/journal.pone.0028740)

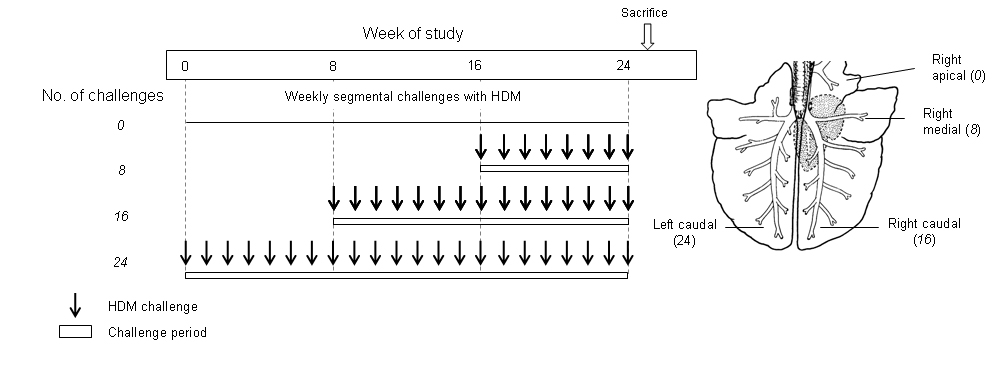

Supplement: Figure S1 — Schematic diagram showing the protocol for segmental allergen challenge in four spatially separate lung segments. (a) The right apical, right medial, right caudal and left caudal segments received 0, 8, 16 and 24 weekly infusions of 1 mg house dust mite allergen in 5 mL of phosphate buffered saline. (b) Schematic diagram of a sheep lung showing the location of each lung segment. (TIF) [file pone.0028740.s001.tif]

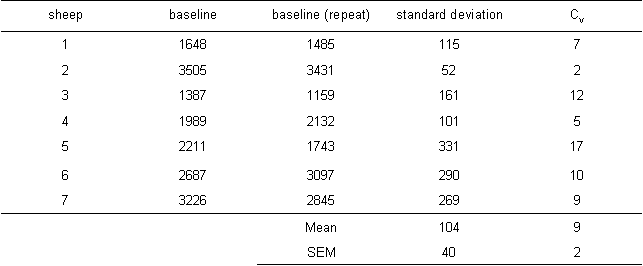

Supplement: Table S1 — Repeated measures of resting peripheral resistance. Repeated measurements of the resting peripheral resistance (Rp) in individual sheep collected two weeks apart. Units are expressed in cmH2O/L/min. Repeated measures were collected two weeks apart. Cv – coefficient of variation. SEM – standard error of the mean. (TIF) [file pone.0028740.s002.tif]
